# Object Detection Based on Template Matching through Use of Best-So-Far ABC

**DOI:** 10.1155/2014/919406

**Published:** 2014-04-09

**Authors:** Anan Banharnsakun, Supannee Tanathong

**Affiliations:** ^1^Laboratory for Computational Intelligence, Faculty of Engineering at Sriracha, Kasetsart University Sriracha Campus, Chonburi 20230, Thailand; ^2^Laboratory for Sensor and Modeling, Department of Geoinformatics, University of Seoul, Seoul 130-743, Republic of Korea

## Abstract

Best-so-far ABC is a modified version of the artificial bee colony (ABC) algorithm used for optimization tasks. This algorithm is one of the swarm intelligence (SI) algorithms proposed in recent literature, in which the results demonstrated that the best-so-far ABC can produce higher quality solutions with faster convergence than either the ordinary ABC or the current state-of-the-art ABC-based algorithm. In this work, we aim to apply the best-so-far ABC-based approach for object detection based on template matching by using the difference between the RGB level histograms corresponding to the target object and the template object as the objective function. Results confirm that the proposed method was successful in both detecting objects and optimizing the time used to reach the solution.

## 1. Introduction

Template matching is a technique in computer vision used for finding a subimage of a target image which matches a template image. This technique is widely used in object detection fields such as surveillance [1], vehicle tracking [2], robotics [3], medical imaging [4], and manufacturing [5]. Generally, template matching approaches can be categorized into two groups, the first based on the level histogram method and the second based on the feature extraction method. Here, the focus is on the former group because the relevant methods of the level histogram are simple to operate, and its accuracy and error estimates have already undergone quantitative analysis and the research results can be found in the previous literature [6–9]. However, this method also requires extensive computational cost since the matching process involves moving the template image to all possible positions in a larger target image and computing a numerical index that indicates how well the template matches the image in that position. This problem is thus considered as an optimization problem. The algorithms based on swarm intelligence approach have been considered as a way to alleviate the drawback of the long processing time in this problem in recent works [10–17].

Swarm intelligence [18] is a metaheuristic method in the field of artificial intelligence, used to solve optimization problems. It is based on the collective behavior of social insects, flocks of birds, or schools of fish. These animals can solve complex tasks without having a centralized control unit. The well-known algorithms in swarm intelligence domain that have emerged in recent years include ant colony optimization (ACO) [19], based on the foraging behavior of ants, particle swarm optimization (PSO) [20], based on the behaviors of bird flocks and fish schools, and artificial bee colony (ABC) [21], based on bee foraging behavior.

The properties of ACO, which are useful in finding global optima, and the normalized product correlation (Nprod) of images, which is adopted as a similarity measure, were introduced by Li et al. [10] to estimate the matching position between the template image and the reference image of the same scene. Yao et al. [11] presented an approach to the adaptive template matching based on an improved ACO algorithm by using the coarse-fine searching method to make the matching result more accurate and robust. Wadhwa and Lien [12] employed the ACO algorithm to solve the object recognition problem in a robot material handling system. The normalized cross-correlation (NCC) function was used as an objection function in the optimization procedure. A hybrid algorithm of PSO and differential evolution (DE) was proposed by Zhao et al. [13] in order to improve the local search ability in the gray scale matching process. Yan et al. [14] proposed a model-matching algorithm based on the gray of the image for supporting the process of vision guided autonomous underwater vehicle (AUV) docking. The model employed the Quanta-PSO to find the best matching point and used the gray absolute correlative degree of the target image and template image as the fitness function. To prevent any errors that may occur when the environment to be tracked becomes too diverse, the PSO based approach for multiple objects tracking by using histogram matching was presented by Hsu and Dai [15]. Chidambaram and Lopes [16] applied the ABC to object recognition in digital images. The absolute sum of the difference of intensity between pixels of the target image and the template image was considered as the dissimilarity function instead of using similarity measures. The ABC algorithm with edge potential function (EPF) was proposed by Xu and Duan [17] to accomplish the target recognition task for aircraft. This hybrid method took advantage of the accuracy and stability of EPF in target shape recognition, and the ABC algorithm was adopted to optimize the matching parameters.

In this paper, we propose the use of the best-so-far ABC-based approach for object detection based on template matching by using the difference between the RGB level histogram corresponding to the target object and the template object. The research aims to improve the solution quality, which is measured based on the accuracy in detecting the target object, and to optimize the time used to reach the solution.

The paper is organized as follows.  Section 2 describes the template matching function.  Section 3 presents a brief concept of the best-so-far ABC algorithm.  Section 4 proposes the use of the best-so-far ABC in object detection.  Section 5 presents the experiments and results.  Section 6 summarizes the conclusions of the work.

## 2. Template Matching Function

The difference between the RGB level histograms corresponding to the target object and the template object is presented as the matching measure function in this work. The histograms of both the target and template images are calculated from a plot of each independent channel of the triplet RGB level values versus the number of pixels for each tonal value.

Let *HI*_*i*_^*x*^ be the histogram of the target object, *HJ*_*i*_^*x*^ the histogram of the template object, where *i* = 0 to 255 stands for the level values of each channel of the triplet RGB, and *x* = {*R*, *G*, *B*}.

Thus

(1) HIix=∑m=rM∑n=cNgm,n,where gm,n={1if  value  at  (m,n)  in  channel  x  equal  to  i0otherwise,HJix=∑m=0M ∑n=0Ngm,n,where gm,n={1if  value  at  (m,n)  in  channel  x  equal  to  i0otherwise,

where (*r*, *c*) denotes the planar coordinates of the top left corner of the template image with size *M* × *N* relative to the target image. If the target image has a size of *A* × *B*, then 0 ≤ *r* ≤ *A* − *M* and 0 ≤ *c* ≤ *B* − *N*.

**Figure 1:**
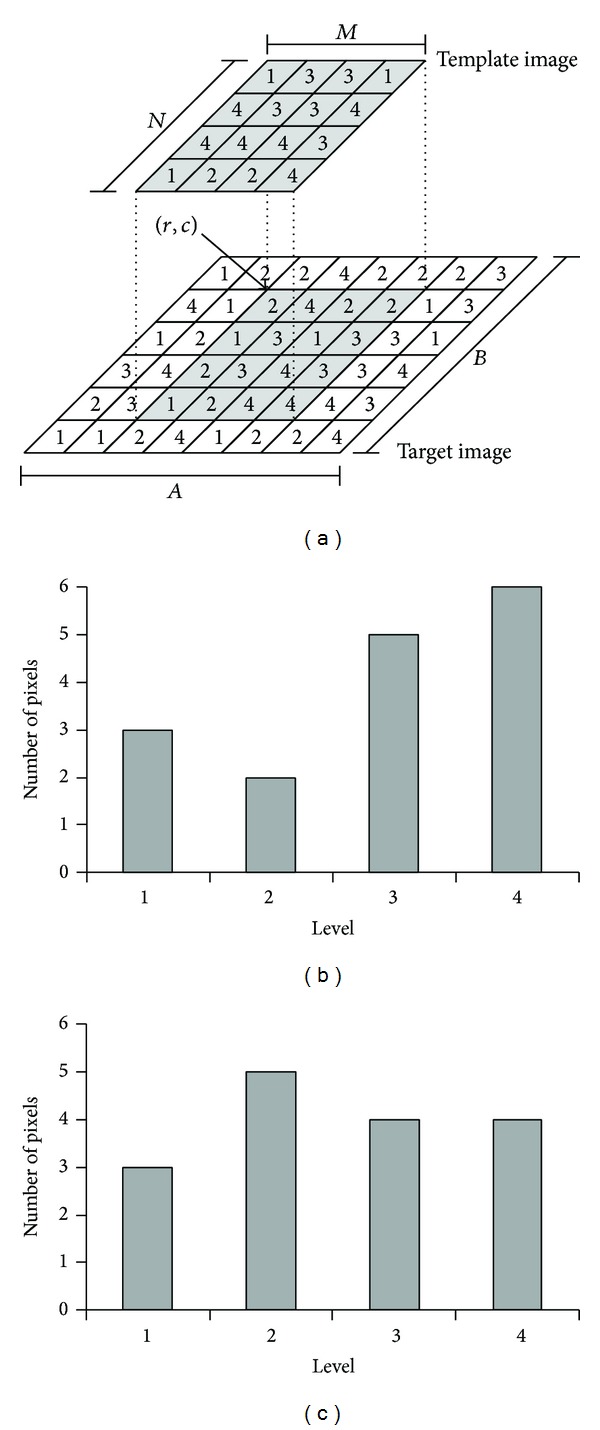
(a) The sample value of each pixel in the target and template images; (b) the histogram obtained from the template image; (c) the histogram obtained from the target image.

More clearly, Figure 1 illustrates how to obtain the image histogram from the sample target with a size of 8 × 6 pixels and template with a size of 4 × 4 pixels where (*r*, *c*) = (3,2).  Figure 1(a) presents the value of each pixel in each image. Figures 1(b) and 1(c) show the histogram of these sample images.

Based on the assumption that the matching result between the histogram of the target object and the histogram of the template object should be improved if we consider all color bands (red, green, and blue) instead of converting them to gray band presented by Hsu and Dai [15], we define the equation used to calculate the difference between each color level histogram corresponding to the target object and the template object as follows.

For red band,

(2) $$\begin{array}{c} D_{R}=\overset{255}{\underset{i}{\sum }}{\left(HI_{i}^{R}-HJ_{i}^{R}\right)}^{2}. \end{array}$$

For green band,

(3) $$\begin{array}{c} D_{G}=\overset{255}{\underset{i}{\sum }}{\left(HI_{i}^{G}-HJ_{i}^{G}\right)}^{2}. \end{array}$$

For blue band,

(4) $$\begin{array}{c} D_{B}=\overset{255}{\underset{i}{\sum }}{\left(HI_{i}^{B}-HJ_{i}^{B}\right)}^{2}. \end{array}$$

Finally, we define the difference between the RGB level histogram of the target object and template object (*D*) as shown in (5) by calculating the summation of the difference between each color level histogram corresponding to the target object and the template object obtained from (2) to (4) and normalizing it by using the summation of their square root values. The best matching image can be determined by finding the minimum value of this function. Consider

(5) D=(∑i255(HIiR−HJiR)2+∑i255(HIiG−HJiG)2   +∑i255(HIiB−HJiB)2)  ×(∑i255(HIiR−HJiR)2+∑i255(HIiG−HJiG)2    +∑i255(HIiB−HJiB)2)−1.

## 3. The Best-So-Far ABC Algorithm Concept

Although the activities of exploitation and exploration are well balanced and help to mitigate both stagnation and premature convergence in the ABC algorithm, the convergence speed is still an issue in some situations. To enhance the exploitation and exploration processes, three major changes made by introducing the best-so-far method (BSF), an adjustable search radius (ASR), and an objective-value-based comparison method (OBC) were presented by Banharnsakun et al. [22]. The experimental results have demonstrated that the best-so-far ABC is able to produce higher quality solutions with faster convergence than the original ABC and other state-of-the-art heuristic-based algorithms [22–25]. To better understand the best-so-far ABC concept, a brief description of these three modifications of the best-so-far ABC is presented in the next section.

### 3.1. The Best-So-Far Method (BSF)

In the original ABC algorithm [1], each onlooker bee selects a food source based on a probability that varies according to the fitness function explored by a single employed bee. Then, the new candidate solutions are generated by updating the onlooker solutions as shown in

(6) $$\begin{array}{c} v_{ij}=x_{ij}+\phi_{ij}\left(x_{ij}-x_{kj}\right). \end{array}$$

In (6), *v*_*ij*_ is a new feasible solution that is modified from its previous solution value (*x*_*ij*_) based on a comparison with the randomly selected position from its neighboring solution (*x*_*kj*_). *ϕ*_*ij*_ is a random number between [−1,1] which is used to adjust the old solution to become a new solution in the next iteration. *k* ∈ {1,2, 3,…, SN}∧*k* ≠ *i* and *j* ∈ {1,2, 3,…, *D*} are randomly chosen indexes. The difference between *x*_*ij*_ and *x*_*kj*_ is a difference of position in a particular dimension. However, changing only one dimension of the solution *x*_*i*_ in the original ABC results in a slow convergence rate.

In the best-so-far method, all onlooker bees use existing information from all employed bees to make a decision on a new candidate food source. Thus, the onlookers can compare information from all candidate sources and are able to select the best-so-far position. The new method used to calculate a candidate food source is shown in

(7) $$\begin{array}{c} v_{id}=x_{ij}+\Phi f_{b}\left(x_{ij}-x_{bj}\right), \end{array}$$

where *v*_*id*_ is the new candidate food source for onlooker bee position *i* dimension *d*, *d* = 1,2, 3,…, *D*, *x*_*ij*_ is the selected food source position *i* in a selected dimension *j*, Φ is a random number between − 1 and 1, *f*_*b*_ is the fitness value of the best food source so far, and *x*_*bj*_ is the best food source so far in a selected dimension *j*.

### 3.2. The Adjustable Search Radius (ASR)

Although the best-so-far method can increase the local search ability compared to the original ABC algorithm, the solution is easily entrapped in a local optimum. In order to resolve this issue, improvement of both exploitation and exploration based on a global search ability of the scout bee has been introduced.

In the best-so-far ABC, the scout bee will randomly generate a new food source by using (8) whenever the solution stagnates in the local optimum as follows:

(8) $$\begin{array}{c} v_{ij}=x_{ij}+\phi_{ij}\left[\omega_{\max}-\frac{\text{iteration}}{\text{MCN}}\left(\omega_{\max}-\omega_{\min}\right)\right]x_{ij}, \end{array}$$

where *v*_*ij*_ is a new feasible solution of a scout bee that is modified from the current position of an abandoned food source (*x*_*ij*_) and *ϕ*_*ij*_ is a random number between [−1,1]. The values of *ω*_max⁡_ and *ω*_min⁡_ represent the maximum and minimum percentage of the position adjustment for the scout bee. The values of *ω*_max⁡_ and *ω*_min⁡_ are fixed to 1 and 0.2, respectively. These parameters were chosen by the experimenter. With these selected values, the adjustment of scout bee's position based on its current position will linearly decrease from 100 percent to 20 percent in each experiment round; that is, a scout bee will utilize the exploration process in the early part of the process and will employ the exploitation process by using existing information of the solution in the later steps.

### 3.3. The Objective-Value-Based Comparison Method (OBC)

Basically, the comparison of the new solution and the old solution is performed by the fitness value. If the fitness of the new solution is better than the fitness of the old solution, we select the new one and ignore the old solution. The fitness value can be obtained from the following:

(9) $$\begin{array}{c} \text{Fitness}\left(f\left(x\right)\right)=\{\begin{array}{cc} \frac{1}{1+f\left(x\right)} & \text{if}\;\;f\left(x\right)\geq 0\\ 1+\left|f\left(x\right)\right| & \text{if}\;\;f\left(x\right)<0. \end{array} \end{array}$$

Based on (9), we can see that when *f*(*x*) is larger than zero but has a very small value, for example, 1*E* − 20, the fitness value of equation 1/(1 + 1*E* − 20) is rounded up to be 1 (1*E* − 20 is ignored). This will lead the fitness of all solutions to become equal to 1 in the later iterations. In other words, there is no difference between the fitness values that are equal to 1/(1 + 1*E* − 20) and 1/(1 + 1*E* − 120). Thus, a new solution that gives a better fitness value than the old solution will be ignored and the solution will stagnate at the old solution. In order to solve this issue, the objective value of function is directly used to compare and to select between the old solution and the new solution in each iteration.

## 4. Using the Best-So-Far ABC in Object Detection

**Figure 2:**
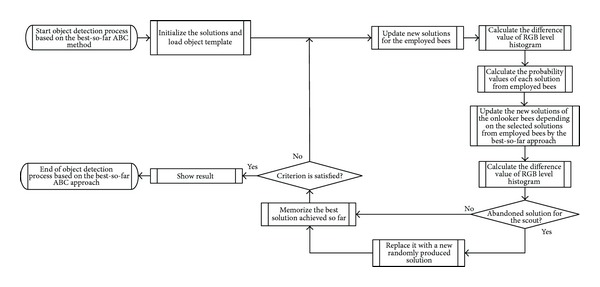
Object detection based on the best-so-far ABC method.

The best-so-far ABC algorithm was applied to the object detection problem based on the template matching described in Section 2. The goal is to find a global optimization of the similarity measure. In other words, we try to find the possible solution (*r*, *c*), which represents the planar coordinates of the top left corner of the template image relative to the target image that minimize the difference value of RGB level histogram in (2). The applied algorithm is illustrated in Figure 2.

In Figure 2, initial solutions (*r*, *c*) are generated. These parameters are treated as the food sources for the employed bees. Each solution is used to move the template image to all possible positions in the target image. Then, we calculate the difference value of RGB level histogram between the target image and the moved template image. The onlooker bees will then select the solutions that produce a lower difference value of RGB level histogram and update those solutions based on the best-so-far method. The process will be repeated until the difference value of RGB level histogram reaches a zero value or the number of iterations equals the MCN. Solutions that cannot decrease the difference value of RGB level histogram within a certain period will be abandoned and new solutions will be regenerated by the scout bee.

## 5. Experiment Setting and Results

We validated the performance of the proposed technique compared with previous works including the PSO with RGB histogram method, the PSO with gray histogram method [15], and the PSO with normalized cross-correlation (NCC) method [26]. We aimed at comparing and evaluating the solution quality obtained from our proposed approach and other aforementioned methods from the perspectives of the detection accuracy and the time used to reach the solution.

Once again, the objective function of the object detection based template matching in this work is the difference value of RGB level histogram (*D*). *D* is used to measure the dissimilarity of the target image and the template images after matching. Thus, the lower the *D* value, the more accurate the matching process.

**Figure 3:**
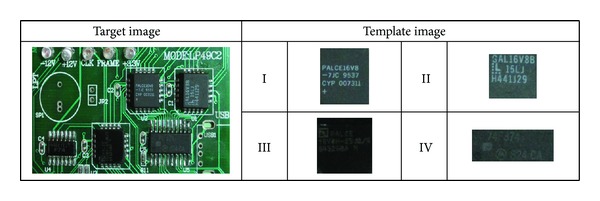
Target image and four sample template images used in the experiments.

All methods in this experiment were programmed in C++ and all experiments were run on a PC with an Intel Core i7 CPU, 2.8 GHz and 16 GB memory. For the proposed method, the numbers of employed and onlooker bees were set to 10. The values of *ω*_max⁡_ and *ω*_min⁡_ were set to 1 and 0.2, respectively, and the number of iterations (MCN) was set to 70. For the parameter setting of the PSO with gray histogram and the PSO with NCC methods, the number of particles was set to 20, the parameters used in PSO were defined as *c*_1_ = *c*_2_ = 2, *ω* = 0.7, and the number of iterations was set to 250. In our experiments, we used four sample template images as shown in Figure 3. For each template image, the experiments were repeated 30 times with different random seeds.

**Figure 4:**
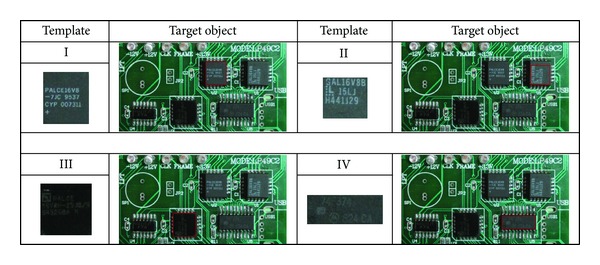
Detected object results by the best-so-far ABC with RGB histogram.

**Figure 5:**
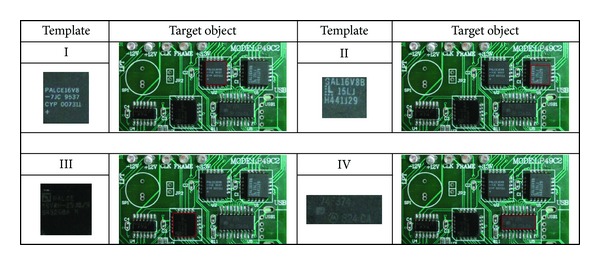
Detected object results by the PSO with RGB histogram.

**Figure 6:**
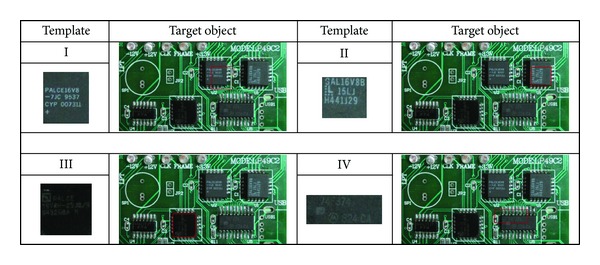
Detected object results by the PSO with gray histogram.

**Figure 7:**
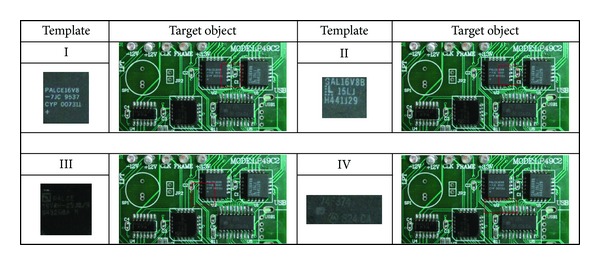
Detected object results by the PSO with NCC.

The results obtained from the best-so-far ABC with RGB histogram, the PSO with gray histogram, and the PSO with NCC methods are listed in Table 1 and illustrated in Figures 4, 5, 6, and 7, respectively. The “% accuracy” column shows the percentage of the total number of the correct detections from 30 experiments and the “time used” column shows the average time used in units of seconds to find the object. The “% accuracy” can be calculated by

(10) %Accuracy=(Total  number  of  correct  detectionsfrom  30  experiments)×(30)−1×100.

The criterion used to judge the detection result from each experiment as a failure or success can be obtained from the condition as follows: if the possible (*r*, *c*) solution found by the algorithm is more than 5 pixels far from the actual (*r*, *c*) solution, it is considered as a failure; otherwise, it is considered as a success.

From Table 1, it can be seen that the best-so-far ABC with RGB histogram generates better results in terms of the detection accuracy and the time used than the PSO with RGB histogram, the PSO with gray histogram, and the PSO with NCC. Both the best-so-far ABC and the PSO with RGB histogram and the PSO with gray histogram were able to detect all of the 4 template images, whereas the PSO with NCC was able to detect only 2 among 4 of the template images. However, both the best-so-far ABC and the PSO with RGB histogram algorithms were able to detect the object with a 100% accuracy rate, whereas the PSO with gray histogram and the PSO with NCC algorithms were able to detect it only 92% and 14% of the time, respectively. Moreover, the best-so-far ABC with RGB histogram technique continued to give good results in terms of the time used compared with the PSO techniques. The average amount of time used for the best-so-far ABC with RGB histogram technique was 0.680 seconds, whereas the PSO with RGB histogram, the PSO with gray histogram, and the PSO with NCC techniques took 2.375, 0.947, and 14.320 seconds, respectively.

These results indicate that the best-so-far ABC with RGB histogram method solutions converged to an optimal solution more quickly than other aforementioned methods in all template images. Comparing between the best-so-far ABC and the PSO based on the same RGB histogram matching function, a maximum runtime improvement of 73% was found in the experiment with template image II and a minimum of 70% was found with template image I. The average runtime improvement for all template images was 71%. In a comparison between the best-so-far ABC with RGB histogram and the PSO with gray histogram methods, a maximum runtime improvement of 29% was found in the experiment with template image III and a minimum of 27% was found with template image IV. The average runtime improvement for all template images was 28%. Comparing between the best-so-far ABC with RGB histogram and the PSO with NCC methods, the average runtime improvement for all template images was 95%.

In summary, even though all of the algorithms presented have a similar search process based on metaheuristic methods, the best-so-far ABC method was able to provide better results than the PSO methods. The best-so-far ABC method makes use of both exploitation and exploration in its search process, while the PSO methods only have exploitation. The exploitation is handled by employed bees and onlooker bees, while the exploration is maintained by scout bees in the best-so-far ABC method. If some solutions become trapped at any local optima, the scout bees will try to randomly search for a new solution again. Moreover, the difference between the RGB level histograms corresponding to the target object and the template object proposed in this work as the matching measure function also helps the algorithm to evaluate the matching quality more precisely than the gray histogram method and to calculate the matching result more quickly than the NCC approach. The object detection based on the RGB histogram matching by using the best-so-far ABC method can thus achieve an accuracy rate of 100% and use a low amount of computational time compared to both the PSO with the gray histogram and the PSO with NCC methods.

## 6. Conclusions

Object detection based on template matching by using the best-so-far ABC was proposed and the difference between the RGB level histograms corresponding to the target object and the template object was presented as the matching measure function in this work.

The performance of the best-so-far ABC with RGB histogram method was then compared with previous works including the PSO with RGB histogram method, the PSO with gray histogram method, and the PSO with normalized cross-correlation (NCC) method. The detection accuracy and the computational time used for detecting objects were set as the objectives in this detection process.

The results obtained from our proposed method show that the best-so-far ABC with RGB histogram can detect the object more effectively than other aforementioned approaches. Thus, we can conclude that the best-so-far ABC with RGB histogram image matching is highly efficient from the perspective of both solution quality and algorithm performance in a computer vision system for real-world object detection in images.

## Figures and Tables

**Table 1:** Comparison of results between the best-so-far ABC and other approaches.

Template image	The best-so-far ABC with RGB histogram		PSO with RGB histogram		PSO with gray histogram [15]		PSO with NCC [26]	
	% accuracy	Time used (s)	% accuracy	Time used (s)	% accuracy	Time used (s)	% accuracy	Time used (s)
I	100.00	0.719	100.00	2.430	90.00	1.001	40.00	15.052
II	100.00	0.557	100.00	2.113	93.33	0.774	0.00	11.713
III	100.00	0.775	100.00	2.636	96.67	1.098	0.00	16.720
IV	100.00	0.669	100.00	2.320	86.67	0.915	36.67	13.795

Average	100.00	0.680	100.00	2.375	91.668	0.947	19.168	14.320
